# Cerebrospinal fluid biomarkers for Alzheimer disease and subcortical axonal damage in 5,542 clinical samples

**DOI:** 10.1186/alzrt212

**Published:** 2013-10-14

**Authors:** Tobias Skillbäck, Henrik Zetterberg, Kaj Blennow, Niklas Mattsson

**Affiliations:** 1 Clinical Neurochemistry Laboratory, Institute of Neuroscience and Physiology, Department of Neurochemistry, Sahlgrenska University Hospital/Mölndal, Mölndal, Sweden; 2 UCL Institute of Neurology, Queen Square, London, WC1N 3BG, UK; 3 Department of Veterans Affairs Medical Center, Center for Imaging of Neurodegenerative Diseases, San Francisco, CA, USA

## Abstract

**Introduction:**

The neuronal loss in Alzheimer disease (AD) has been described to affect grey matter in the cerebral cortex. However, in the elderly, AD pathology is likely to occur together with subcortical axonal degeneration on the basis of cerebrovascular disease. Therefore, we hypothesized that biomarkers for AD and subcortical axonal degeneration would correlate in patients undergoing testing for dementia biomarkers, particularly in older age groups.

**Methods:**

We performed correlation and cluster analyses of cerebrospinal fluid (CSF) biomarker data from 5,542 CSF samples analyzed in our routine clinical neurochemistry laboratory in 2010 through 2012 for the established CSF AD biomarkers total tau (T-tau), phosphorylated-tau (P-tau), amyloid β1-42 (Aβ42), and for neurofilament light (NFL), which is a protein expressed in large-caliber myelinated axons, the CSF levels of which correlate with subcortical axonal injury.

**Results:**

Aβ42, T-tau, and P-tau correlated with NFL. By cluster analysis, we found a bimodal data distribution in which a group with a low Aβ42/P-tau ratio (suggesting AD pathology) had high levels of NFL. High levels of NFL also correlated with the presence of an AD biomarker pattern defined by Aβ42/P-tau and T-tau. Only 29% of those with an AD biomarker signature had normal NFL levels. Age was a possible confounding factor for the associations between NFL and established AD biomarkers, but in a logistic regression analysis, both age and NFL independently predicted the AD biomarker pattern.

**Conclusions:**

The association between an AD-like signature using the established biomarkers Aβ42, T-tau, and P-tau with increased levels of NFL provides *in vivo* evidence of an association between AD and subcortical axonal degeneration in this uniquely large dataset of CSF samples tested for dementia biomarkers.

## Introduction

A growing interest exists in the role of mixed pathologies for development of symptomatic Alzheimer disease (AD). The canonic brain hallmarks of AD are extracellular β-amyloid (Aβ) deposits and intracellular tangles composed of tau proteins, but the correlations between these findings and dementia symptoms decline with increasing age [1]. Although pathologic Aβ metabolism is clearly the initiating event in autosomal dominant AD, sporadic AD may be a more heterogeneous etiology, with, for example, cerebrovascular pathology contributing to symptoms in some patients [2]. A large overlap in pathology is noted between elderly AD patients and individuals dying of other causes [3-5], which might be explained by combined effects of Alzheimer-type and cerebrovascular pathology in the form of white-matter lesions actually leading to dementia in elderly AD patients [6]. This is supported by neuropathologic studies showing increased frequency of mixed pathologies rather than pure AD pathology in dementia patients in older age groups [7].

Cerebrospinal fluid (CSF) biomarkers have become increasingly important in dementia research and have been included in research diagnostic criteria for AD [8-10]. The most well-established CSF AD biomarkers are the 42-amino-acid isoform of Aβ (Aβ42), total-tau (T-tau), and phosphorylated tau (P-tau), which are believed to reflect the presence of amyloid plaque deposition, axonal degeneration, and neurofibrillary tangles, respectively [11]. Another CSF biomarker for neuroaxonal injury is neurofilament light (NFL) protein. NFL is expressed predominantly in large-caliber myelinated axons [12], and its CSF levels correlate with white-matter lesions and other injuries to subcortical brain regions [13-18]. NFL is often normal in clinically pure AD [19] but abnormal in the presence of vascular pathology (for example, small-vessel disease [20], which mainly affects subcortical brain regions). NFL is also elevated in several other pathologic conditions, such as frontotemporal dementia (FTD) [21,22], MS [23], idiopathic normal-pressure hydrocephalus [24], ALS [25], and various CNS infections [26-28]. Mutations in the NFL gene cause Charcot-Marie-Tooth disease [29].

Here, our main goal was to explore *in vivo* relations between pathologic hallmarks of AD and subcortical axonal damage, by using CSF biomarkers. Because the influence of vascular pathology and other causes of injury to subcortical brain regions in the general AD population may be underestimated in small cohorts with selected AD patients, we performed a study of CSF biomarkers in a uniquely large sample set from our clinical routine practice. This included mainly samples from memory clinics, geriatric clinics, and neurology clinics. In this unselected material, we hypothesized that a positive CSF AD biomarker signature, with reduced Aβ42 and elevated T-tau and P-tau, would often be seen in combination with elevated NFL, because of the presence of mixed pathology, particularly in the older age groups. We assumed that the main cause of elevated NFL was subcortical vascular disease, but it is possible that NFL may also be elevated because of increased axonal loss in general, or secondary to amyloid angiopathy, which may accompany and correlate with AD pathology, and lead to cerebrovascular pathology.

## Methods

This was a cross-sectional study, using archived data on CSF Aβ42, T-tau, P-tau, and NFL measurements from January 1, 2010, to June 1, 2012, extracted from the database of clinical routine test results at the Mölndal site of the Sahlgrenska University Hospital, Sweden. All test results were obtained for diagnostic purposes. The inclusion criteria were that patients were older than 30 years at sampling, and that all four CSF analyses had been requested by the clinician. The majority of the samples were ordered from geriatric clinics, neurology clinics, and memory clinics. For patients tested more than once during the study period (*N* = 126), only the first sample was used. In all, the study included 5,442 samples. Because this was a registry study, the concept of informed consent was not applicable. The laboratory receives samples from many different clinical sites in Sweden, so we did not have access to medical records, CSF cell counts, or total protein levels. This makes us unable to rule out cases of infectious, inflammatory, or acute cerebrovascular disease in this cohort.

### Biochemical measurements

CSF Aβ42, T-tau, and P-tau were measured by using enzyme-linked immunosorbent assay (ELISA) assays (INNOTEST β-amyloid [1–42], hTau Ag, and Phospho-tau [181P]; Innogenetics) as previously described [30,31]. The between-assay coefficients of variation (CV) for the Aβ42, T-tau, and P-tau tests were 13.4%, 11.3%, and 9.70%, respectively (as determined by internal control samples during the entire study period). CSF NFL was measured with a novel, sensitive sandwich ELISA method (NF-light ELISA kit; UmanDiagnostics AB, Umeå, Sweden), as described by the manufacturer. The lower limit of quantification for this assay was 50 ng/L. The between-assay CV for the NFL assays was 14.0%. For further details on these measurements, see Appendix A.

For the AD biomarkers, we used cut-offs previously generated at our laboratory, in a study with long follow-up time of early-stage AD patients, in which the combination of T-tau and Aβ42/P-tau ratio had 95% sensitivity and 87% specificity [32]. With these cut-offs (T-tau > 350 ng/L; Aβ42/P-tau < 6.5), our dataset was divided into groups of sample profiles matching the AD biochemical profile (26.9%) or not (73.1%).

For NFL, we defined a threshold value for subcortical axonal degeneration by using an independent dataset on 108 CSF samples from clinically examined neurologically healthy volunteers (age median, 38 years; range, 18 to 76 years). The 95^th^ percentile for CSF NFL in this group gave us a threshold value of 1,400 ng/L. This dataset forms the basis for the clinical normal reference limits for the UmanDiagnostics NFL assay at the Sahlgrenska Mölndal laboratory.

All analyses were performed in clinical routine by board-certified laboratory technicians with procedures accredited by the Swedish Board for Accreditation and Conformity Assessment (SWEDAC). Longitudinal stability in the measurements over years was ascertained by using an elaborate system of internal quality control samples and testing of incoming reagents (Appendix A).

### Variables

The outcomes were relations between NFL levels and the AD biomarkers. In particular, we were interested in the frequency of patients with increased NFL levels among patients with a positive AD biomarker pattern. Age and sex were potential confounders.

### Statistics

Correlations were calculated by using the Spearman rank correlation coefficient, and statistical hypothesis testing was performed by using Pearson χ^2^ test, logistic regression, ANOVA, multiple regression, or Mann–Whitney *U* tests. Outliers in data were identified by the Grubbs test for outliers and excluded from the dataset before analysis. These general statistics and all charts and tables were produced in SPSS version 20 (IBM, New York, NY, USA). Logarithmic transformations were used to fit the significantly skewed NFL data for logistic regression analysis and multiple regression. Figure axes are presented with logarithmic axes where specified in axis title.

Clustering was performed in JMP version 10.0.0 (SAS Institute Inc., Cary, NC, USA) by using the K-means clustering algorithm. K-means clustering is a special case of a general approach called the EM algorithm, where E stands for Expectation (the cluster means in this case), and M stands for Maximization, which in this case means assigning points to the closest clusters. Distance is measured by the squared euclidian distance of each point, assigning each data point to the cluster that yields the least within-cluster sum of squares. To choose the optimal number of clusters, we used the cubic clustering criterion (CCC), in which the possible numbers of clusters are compared with each other in terms of their within-cluster sum of squares [33].

### Ethics

The study was approved by the regional ethics committee at the University of Gothenburg.

## Results

### Dataset description

Demographics are summarized in Table 1. The majority of patients were from 60 to 90 years old (M, 70.1; SD, 11.0), and the sex distribution was even.

**Table 1:** Demographics

**Age group**		**Sex**		**Aβ42**	**T-tau**	**P-tau**	**NFL**
		**F**	**M**				
**All**	**Valid *N***	50.8%	49.2%	5,542	5,542	5,542	5,542
	**Mean (SD)**			602 (286)	504 (953)	60 (33)	3,007 (6,935)
	**Median (range)**			544 (1,725)	360 (32,125)	52 (283)	1,530 (226,920)
**31-40**	**Valid *N***	51.3%	48.7%	78	78	78	78
	**Mean (SD)**			733 (309)	262 (229)	38 (20)	1,974 (6,056)
	**Median (range)**			704 (1,420)	194 (1,325)	35 (122)	460 (45,790)
**41-50**	**Valid *N***	52,.%	47.7%	237	237	237	237
	**Mean (SD)**			739 (304)	302 (494)	39 (18)	1,856 (5,790)
	**Median (range)**			707 (1,725)	210 (6,025)	35 (122)	520 (66,220)
**51-60**	**Valid *n***	52.2%	47.8%	688	688	688	688
	**Mean (SD)**			723 (297)	458 (1,354)	49 (30)	2,788 (8,719)
	**Median (range)**			700 (1,612)	258 (27,525)	42 (206)	870 (100,810)
**61-70**	**Valid *n***	48.9%	51.1%	1,539	1,539	1,539	1,539
	**Mean (SD)**			626 (289)	464 (947)	55 (30)	2,931 (6,609)
	**Median (range)**			580 (1,455)	317 (30,425)	48 (283)	1,280 (89,820)
**71-80**	**Valid *n***	49.6%	50.4%	2,069	2,069	2,069	2,069
	**Mean (SD)**			559 (271)	546 (1,027)	64 (32)	3,026 (7,203)
	**Median (range)**			500 (1,715)	409 (32,125)	57 (240)	1,710 (226,770)
**81-90**	**Valid n**	55.1%	44.9%	905	905	905	905
	**Mean (SD)**			523 (247)	581 (349)	72 (34)	3,573 (5,422)
	**Median (range)**			465 (1,535)	479 (3,493)	65 (235)	2,200 (93,130)
**91+**	**Valid *n***	53.8%	46.2%	26	26	26	26
	**Mean (SD)**			487 (161)	778 (549)	90 (53)	5,605 (8,049)
	**Median (range)**			496 (785)	590 (2,413)	76 (250)	2,845 (42,000)

### CSF AD biomarkers in relation to NFL levels

**Figure 1:**
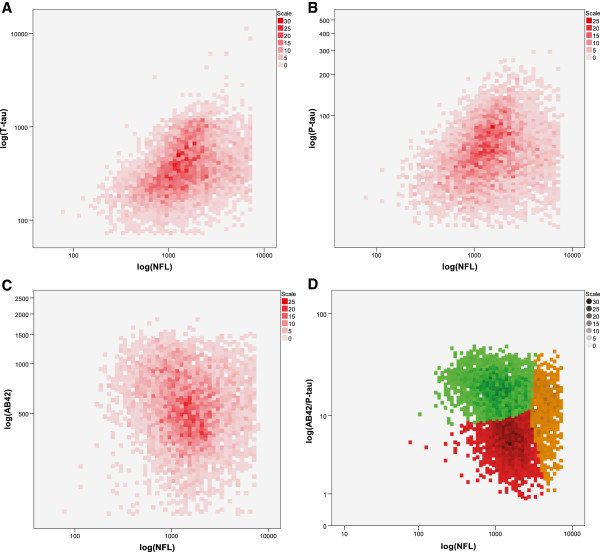
**Relations between NFL and the other markers included in the dataset.** Both T-tau **(A)** and P-tau **(B)** tend to increase with higher levels of NFL, whereas higher levels of Aβ42 **(C)** correlate with lower levels of NFL. For graphic clarity, the x-axes (NFL) were cut at 7,000 ng/L (93% of the tested samples had NFL below this level), but all data points (excluding statistical outliers) were included in the statistical analyses. **(D)** Result of the K-means clustering algorithm applied to NFL and Aβ42/P-tau in our dataset.

We first analyzed the relation between NFL and the other biomarkers in the overall study population (Figure 1). Both T-tau and P-tau had positive correlations with NFL (Figure 1A,B; *R* = 0.416; *P* ≤ 0.001 for T-tau and *r* = 0.231, *P* ≤ 0.001 for P-tau), whereas Aβ42 had an inverse correlation with NFL (Figure 1C; *r* = −0.130; *P* ≤ 0.001).

The combination of the markers Aβ42, P-tau, and NFL had interesting features (Figure 1D). The data distribution contained three separate clusters, with one dense group consisting of samples with a high Aβ42/P-tau ratio in combination with low levels of NFL (green cluster), another group with lower Aβ42/P-tau ratio and higher levels of NFL (red cluster), and a third cluster of outliers with substantially higher levels of NFL (brown cluster). By k-means cluster analysis, we could successfully identify these three groups. By applying CCC, we could confirm that the division into three clusters was the most efficient way to model these data (CCC = 0.62, which was the largest CCC (optimal) in the range of one to 20 clusters [two-cluster CCC = −13.98; four-cluster CCC = −15.23)). The identified clusters showed the following properties: (I, green cluster) high Aβ42/P-tau (median = 18.9; mean = 20.0) and low NFL (median = 1,010; mean = 1,158); (II, red cluster) low Aβ42/P-tau (median = 5.4; mean = 5.5) and high NFL (median = 1,600; mean = 1,697); (III, brown cluster) outliers with very high NFL values (median = 4,440; mean = 4,609) and intermediate Aβ42/P-tau (median = 13.4; mean = 12.1). The age distribution varied across the clusters (M, SD, I: 65.7, 11.5; II: 74.0, 8.6; III: 73.2, 10.0), with significant differences when tested with one-way ANOVA (*F*(2, 5,154) = 413, 193; *P* < 0.001).

### Biochemical AD criteria versus NFL levels

**Figure 2:**
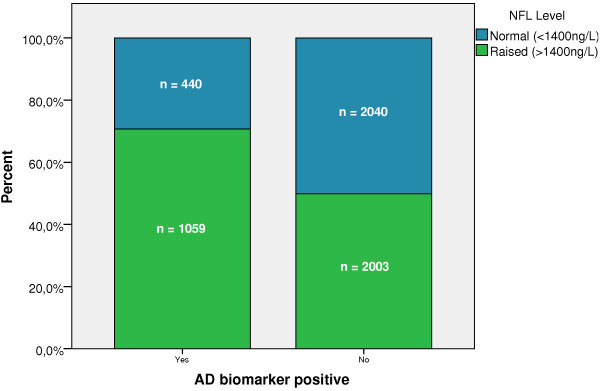
**More patients in the group with positive AD biomarker pattern also display increased NFL levels.** The chart shows the percentage relations between the prevalence of normal or increased NFL levels in those who fulfill AD biomarker criteria or not.

To search further for associations between AD and subcortical pathology, we classified the study subjects by using NFL as a marker of subcortical axonal degeneration, and Aβ and tau markers for AD, by cut-offs, as explained earlier (Figure 2). The relative number of NFL-positive patients was significantly higher in the group of AD biomarker-positive patients than in the AD biomarker-negative group (χ^2^ = 197.0; *P* ≤ 0.001).

### CSF biomarker patterns in different ages

Age is a strong risk factor for both AD and cerebrovascular pathology, which is a main cause of subcortical axonal degeneration, and a possible confounder for the relation between the AD biochemical profile and elevated NFL. We therefore explored the relation between age and biomarkers in detail.

**Figure 3:**
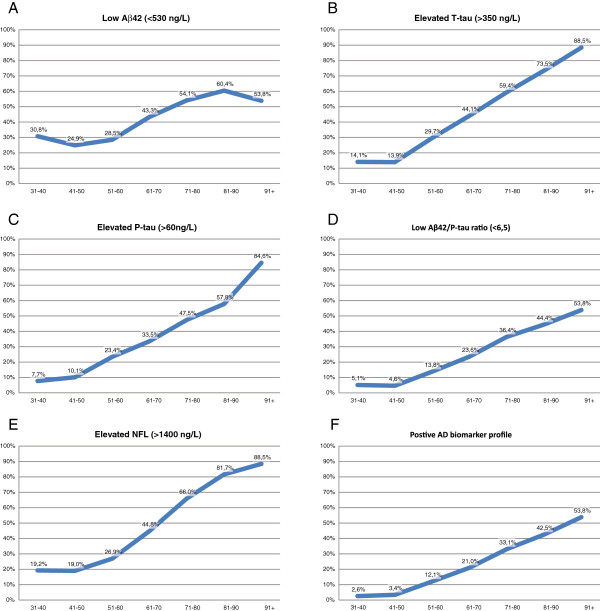
**Pathologic levels of biomarkers in different age groups. (A-D)** Shares of subjects with presumed pathologic levels of the CSF biomarkers Aβ42, T-tau, P-tau, and Aβ42/P-tau-ratio, in that order. For NFL **(E)**, we defined a cut-off by using an independent dataset on 108 CSF samples from clinically examined neurologically healthy adults (age median, 38 years; range, 18 to 76 years). For the AD biomarkers **(F)**, we used cut-offs previously generated at our laboratory, in a study with long follow-up time of early-stage AD patients.

First, pathologic levels of all the biomarkers were explored in the different age groups (Figure 3). The frequency of pathologic levels of Aβ42 was about 30% in the youngest cohort, started to increase in subjects from about 60 years of age, and plateaued at about 60% in patients older than 80 years (Figure 3A). In contrast, the frequency of pathologic levels of T-tau was only about 15% in the youngest cohort, and increased linearly in subjects from about age 40 years, reaching 90% in the oldest cohort (Figure 3B). P-tau had a similar pattern, but with an even more dramatic increase in the oldest old (Figure 3C). Pathologic levels of the Aβ42/P-tau ratio were seen in only about 5% of subjects in the youngest cohort and increased linearly in subjects from about 40 years of age, reaching 55% in the oldest cohort (Figure 3D). The pathologic combination of all three AD biomarkers (AD biomarker profile) had a similar pattern (Figure 3E). Pathologic levels of NFL were seen in about 20% in the youngest cohort and showed a sigmoidal increase, reaching about 90% in the oldest cohort (Figure 3F).

**Figure 4:**
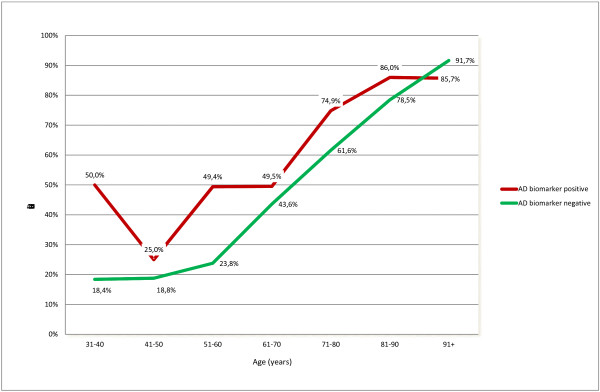
**The prevalence of subjects with increased NFL levels in patient groups who fulfill AD biomarker criteria or not, and how they develop with age.** Patients with a positive AD biomarker pattern display elevated levels of NFL more often than do patients with a negative AD biomarker pattern, but the gap between the two groups closes with age.

Figure 4 displays the groups of AD biomarker-positive and -negative patients, and the prevalence of increased NFL levels in different age groups. It was more common among AD biomarker-positive patients to have low NFL levels in younger than in older patients (although the number of young patients with the AD biochemical profile was small), but the fraction of NFL-positive subjects increased with age, in both the AD-positive and AD-negative groups. To adjust for the effect of age, we did a logistic regression analysis with age and NFL as predictors of the AD biomarker profile (dichotomized as positive or negative). Both age (B = −0.063; *P* ≤ 0.001; exp(B) = 0.939) and log(NFL) (B = −0.685; *P* ≤ 0.001, exp(B) = 0.504) were significant predictors when used as single predictors, and when used together in the model (Age, B = −0.060, *P* < 0.001, exp(B) = 0.941, log(NFL): B = −0.246, *P* = 0.005, Exp(B) = 0.782).

**Figure 5:**
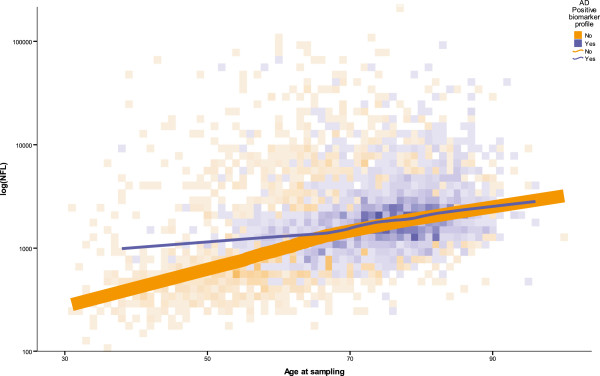
**A plot of NFL levels in patient groups who fulfill AD biomarker criteria or not, and how they develop with age.** Patients with a positive AD biomarker pattern display elevated levels of NFL more often than do patients with a negative AD biomarker pattern, but the gap between the two groups closes with age.

Figure 5 shows NFL values versus age, and LOESS fit lines for the AD biomarker profile- positive and -negative groups. In younger ages, NFL levels were higher in the biomarker-positive group (although the few available AD biomarker-positive young subjects makes these estimates uncertain), but the fitted lines for the positive and negative groups converged around age 65 years.

To verify that the differences in NFL values remained and did not solely rely on the relatively high levels of NFL among younger subjects (as shown in Figures 4 and 5), the data were split into two age categories, with 65 years as break point. A Mann–Whitney *U* test confirmed that the NFL levels in the AD biomarker-positive groups was significantly higher for both age groups (H(1) = 68.6, *P* < 0.001 for ages younger than 65), and (H(1) = 41.53; *P* < 0.001 for ages older than 65).

To assess the importance of different factors in an adjusted model, a multiple linear regression was performed to predict NFL from sex, age, and AD biomarker profile (dichotomized as positive or negative). These variables statistically significantly predicted NFL (*F*(3, 5,538) = 320.31; *P* < 0.001, R_2_ = 0.148). All three variables were independently statistically significant (age (years): β = 0.352, *P* < 0.001; sex (female): β = 0.128; *P* < 0.001; AD-biomarker profile (positive): β = 0.043; *P* = 0.001).

## Discussion

In a uniquely large dataset of clinical routine samples, we tested associations between established CSF biomarkers for AD pathology (Aβ42, T-tau, and P-tau) and a CSF biomarker for subcortical axonal degeneration (NFL). In this unselected population of patients from memory clinics, geriatric clinics, and neurology clinics, we expected to find a large proportion of subjects with positive AD biochemical profile, suggesting the presence of AD, and a large proportion with elevated NFL levels, suggesting the presence of cerebrovascular or other white-matter disease. This was confirmed, but we also found an association between white-matter disease, as reflected by NFL levels and AD, as reflected by Aβ42, T-tau, and P-tau levels. Subjects with a positive AD biomarker profile often also had high NFL levels. Only 29% of the subjects with positive AD biomarker profile had normal NFL levels, suggesting that a pure AD biochemical profile without signs of NFL leakage is unusual in subjects undergoing testing for dementia biomarkers. In contrast, elevated NFL was rare in the AD biomarker-negative group. It should be noted that NFL is believed to be a marker of white-matter disease because it is predominantly expressed in myelinated large-caliber axons [12]. However, other types of injury to such neurons (not only white matter lesions) could also lead to higher NFL levels in CSF.

Cluster analysis showed three clusters in our data; the first large group had high Aβ42/P-tau quota and low NFL (that is, weak biochemical support for either AD or subcortical pathology). The second equally large cluster had low Aβ42/P-tau quota and high NFL (that is, for concomitant AD and subcortical pathology), whereas the third, considerably smaller cluster had the highest levels of NFL and intermediate Aβ42/P-tau quota, suggesting predominant subcortical pathology or other reasons for NFL leakage. This was a cross-sectional study, so we cannot make conclusions on the order of pathologic events underlying these biomarkers, but a possible explanation for the identified biomarker distributions is that AD pathology is strongly associated with vascular pathology, because NFL levels have previously been found to correlate with vascular pathology [15,19,34]. Alternative explanations to our findings may be that the increased NFL levels are caused by high cell loss or increased amyloid angiopathy in AD, or a combination of all these factors.

The cluster with the most normal pattern of Aβ42, P-tau, and NFL (indicating least brain engagement) had the youngest mean age, which may be expected in an unselected group of patients. In a detailed analysis of the relation between biomarkers and age, we observed that the prevalence of high NFL levels increased with age, and we noted that this was seen in patients both with and without the AD biomarker profile. The group with a positive AD biomarker profile had the highest levels of NFL. This difference was greater among the younger patients, and the gap between the groups closed with increasing age, but age and NFL were still independent predictors of the AD biomarker profile in a logistic regression analysis. When dichotomized into subjects younger or older than 65 years, AD biomarker-positive subjects had significantly higher NFL levels in both age groups, tested by using the Mann–Whitney *U* test.

The main strength of this study is its unique size; to our knowledge, it is the largest study ever published on CSF biomarkers. The main study limitation is the limited access to clinical data. The absolute majority of analyses were requested in investigations of cognitive decline. The study population may therefore be considered to represent a group of patients with different degrees of cognitive dysfunction. However, at some centers lumbar puncture and NFL analysis may preferentially be performed in clinically complicated or ambiguous cases, which could bias the population toward rare or mixed brain pathologies, and increase the likelihood of finding atypical biomarker patterns. Because of lack of CSF cell count and total protein data, we cannot exclude that a few patients with inflammatory or infectious conditions were included. However, this limitation is unlikely to have influenced the positive association between high NFL levels and a positive AD biomarker profile, as neuroinflammation might increase NFL levels but not the prevalence of a positive AD biomarker status [35,36]. The lack of clinical data may partly be compensated by the known strong association of positive CSF AD biomarkers with AD pathology [37] and the fact that CSF AD biomarkers are strong predictors of clinical AD in patients undergoing cognitive evaluation [11]. The lack of clinical or imaging data on cerebrovascular disease prevents firm conclusions regarding the cause of NFL elevations. We based the association of elevated CSF NFL with cerebrovascular disease on previous studies [15,19,34].

## Conclusions

Biochemical evidence of AD was most often seen together with elevated NFL levels, which is a biomarker sign compatible with subcortical vascular pathology. However, we acknowledge that several factors may have contributed to NFL elevations in this patient group, including general neuronal death or amyloid angiopathy, which may accompany AD pathology and lead to cerebrovascular pathology. The *in vivo* relation between AD and subcortical axonal degeneration must be further explored in other cohorts with access to clinical, neuroimaging, and fluid biomarker data. The results of this study, if replicated, may be relevant to clinical trials in which drugs developed against AD pathology are evaluated, because we show that patients with an AD biochemical profile are likely to have subcortical axonal degeneration, which may contribute to the symptoms and explain difficulties in obtaining desired drug effects.

### Appendix A

The coefficients of variation (CVs) of the assays for NFL, Aβ42, T-tau, and P-tau were determined by analyzing records of measurements of internal control samples that are routinely carried out at the laboratory at Mölndal at least twice a week. Control samples are kept in frozen aliquots and reused until depleted and then exchanged. Standard deviations of the averages of all measurements from each control sample were calculated, and an average of these standard deviations was used to represent a CV for every analysis.

For NFL, one low and one high control sample was used. The first low control was used from January 2010 until May 2010, whereas the first replaced control was used until January 2011, and the last replaced control was used until June 2012. The three low controls showed standard deviations of 15.15%, 15.24%, and 13.24%, respectively. The first two high controls were used during the same periods as the first two low controls and measured deviations of 14.41% and 16.60%, respectively. The third control was used until May 2012 and had a deviation of 15.32%. The last control was used between May and June 2012 and had a deviation of 7.84%. The average of all these deviations was calculated and resulted in a CV of 14.04%.

For Aβ42, three different low and three different high controls were used during the study. The first low control was used from January 2010 until January 2011; the second one was replaced in May 2012, and the third control was used the remaining time of the test period. The three low controls measured standard deviations of 19.42%, 12.76%, and 12.46%, respectively. The first high control was used from January 2010 until August 2010; the second one, until November 2010; the third, until June 2011; and the last control was used for the remainder of the test period. The high controls measured standard deviations of 10.45%, 13.14%, 12.62%, and 11.54%. The total average of the Aβ42 measurements was calculated as 13.41%.

For P-tau, three different low, and three different high controls were used. The first low control was used from January 2010 until January 2011; the second one was replaced in May 2012; and the third control was used the remaining time of the test period. The three low controls measured standard deviations of 8.60%, 11.68%, and 11.04%, respectively. The first high control was used from January 2010 until November 2010; the second one, until June 2011; and the last control were used for the remainder of the test period. The high controls measured standard deviations of 8.62%, 8.85%, and 9.38%. The total average of the P-tau measurements was calculated to be 9.70%.

For T-tau, three different low, and three different high controls were used. Both the high and low controls were replaced at the same time as the ones for P-tau. The three low controls measured standard deviations of 9.35%, 9.88%, and 9.05%, respectively. The high controls measured standard deviations of 19.15%, 10.73%, and 9.63%. The total average of the T-tau measurements was calculated to be 11.30%.

No measureable longitudinal drift was registered for any of the analyses.

## Abbreviations

AD: Alzheimer disease; Aβ: β-amyloid; Aβ42: Amyloid β1-42; CCC: Cubic clustering criterion; CSF: Cerebrospinal fluid; CV: Coefficient of variation; NFL: Neurofilament light; P-tau: Phosphorylated tau; T-tau: Total tau.

## Competing interests

None of the authors of this article report any disclosures.

## Authors’ contributions

TS performed study design, data analysis, interpretation, and writing of the manuscript draft. HZ contributed to the study concept and design, and to critical revision of the manuscript for important intellectual content. KB added critical revision of the manuscript for important intellectual content. NM performed study supervision, concept, design, interpretation, and critical revision of the manuscript for important intellectual content. All authors read and approved the final manuscript.

## References

[B1] Savva GM Wharton SB Ince PG Forster G Matthews FE Brayne C Medical Research Council Cognitive F, Ageing S Age, neuropathology, and dementia N Engl J Med 2009 5 2302 2309 10.1056/NEJMoa0806142 19474427

[B2] Jellinger KA The pathology of “vascular dementia”: a critical update J Alzheimers Dis 2008 5 107 123 18525132 10.3233/jad-2008-14110

[B3] Morimatsu M Hirai S Muramatsu A Yoshikawa M Senile degenerative brain lesions and dementia J Am Geriatr Soc 1975 5 390 406 50338 10.1111/j.1532-5415.1975.tb00425.x

[B4] Wilcock GK Esiri MM Plaques, tangles and dementia: a quantitative study J Neurol Sci 1982 5 343 356 10.1016/0022-510X(82)90155-1 7175555

[B5] Crystal H Dickson D Fuld P Masur D Scott R Mehler M Masdeu J Kawas C Aronson M Wolfson L Clinico-pathologic studies in dementia: nondemented subjects with pathologically confirmed Alzheimer’s disease Neurology 1988 5 1682 1687 10.1212/WNL.38.11.1682 3185902

[B6] Blennow K Wallin A Clinical heterogeneity of probable Alzheimer’s disease J Geriatr Psychiatry Neurol 1992 5 106 113 1590911 10.1177/002383099200500208

[B7] Jellinger KA Attems J Prevalence of dementia disorders in the oldest-old: an autopsy study Acta Neuropathol 2010 5 421 433 10.1007/s00401-010-0654-5 20204386

[B8] Dubois B Feldman HH Jacova C Cummings JL Dekosky ST Barberger-Gateau P Delacourte A Frisoni G Fox NC Galasko D Gauthier S Hampel H Jicha GA Meguro K O'Brien J Pasquier F Robert P Rossor M Salloway S Sarazin M de Souza LC Stern Y Visser PJ Scheltens P Revising the definition of Alzheimer’s disease: a new lexicon Lancet Neurol 2010 5 1118 1127 10.1016/S1474-4422(10)70223-4 20934914

[B9] Albert MS DeKosky ST Dickson D Dubois B Feldman HH Fox NC Gamst A Holtzman DM Jagust WJ Petersen RC Snyder PJ Carrillo MC Thies B Phelps CH The diagnosis of mild cognitive impairment due to Alzheimer’s disease: recommendations from the National Institute on Aging-Alzheimer’s Association workgroups on diagnostic guidelines for Alzheimer’s disease Alzheimers Dement 2011 5 270 279 10.1016/j.jalz.2011.03.008 21514249 PMC3312027

[B10] McKhann GM Knopman DS Chertkow H Hyman BT Jack CR Jr Kawas CH Klunk WE Koroshetz WJ Manly JJ Mayeux R Mohs RC Morris JC Rossor MN Scheltens P Carrillo MC Thies B Weintraub S Phelps CH The diagnosis of dementia due to Alzheimer’s disease: recommendations from the National Institute on Aging-Alzheimer’s Association workgroups on diagnostic guidelines for Alzheimer’s disease Alzheimers Dement 2011 5 263 269 10.1016/j.jalz.2011.03.005 21514250 PMC3312024

[B11] Blennow K Hampel H Weiner M Zetterberg H Cerebrospinal fluid and plasma biomarkers in Alzheimer disease Nat Rev Neurol 2010 5 131 144 10.1038/nrneurol.2010.4 20157306

[B12] Schlaepfer WW Lynch RG Immunofluorescence studies of neurofilaments in the rat and human peripheral and central nervous system J Cell Biol 1977 5 241 250 10.1083/jcb.74.1.241 326799 PMC2109861

[B13] Zetterberg H Hietala MA Jonsson M Andreasen N Styrud E Karlsson I Edman A Popa C Rasulzada A Wahlund LO Mehta PD Rosengren L Blennow K Wallin A Neurochemical aftermath of amateur boxing Arch Neurol 2006 5 1277 1280 10.1001/archneur.63.9.1277 16966505

[B14] Lycke JN Karlsson JE Andersen O Rosengren LE Neurofilament protein in cerebrospinal fluid: a potential marker of activity in multiple sclerosis J Neurol Neurosurg Psychiatry 1998 5 402 404 10.1136/jnnp.64.3.402 9527161 PMC2170011

[B15] Jonsson M Zetterberg H van Straaten E Lind K Syversen S Edman A Blennow K Rosengren L Pantoni L Inzitari D Wallin A Cerebrospinal fluid biomarkers of white matter lesions: cross-sectional results from the LADIS study Eur J Neurol 2010 5 377 382 10.1111/j.1468-1331.2009.02808.x 19845747

[B16] Tullberg M Blennow K Mansson JE Fredman P Tisell M Wikkelso C Ventricular cerebrospinal fluid neurofilament protein levels decrease in parallel with white matter pathology after shunt surgery in normal pressure hydrocephalus Eur J Neurol 2007 5 248 254 10.1111/j.1468-1331.2006.01553.x 17355543

[B17] Petzold A Keir G Warren J Fox N Rossor MN A systematic review and meta-analysis of CSF neurofilament protein levels as biomarkers in dementia Neurodegener Dis 2007 5 185 194 10.1159/000101843 17596713

[B18] Petzold A Neurofilament phosphoforms: surrogate markers for axonal injury, degeneration and loss J Neurol Sci 2005 5 183 198 10.1016/j.jns.2005.03.015 15896809

[B19] Bjerke M Zetterberg H Edman A Blennow K Wallin A Andreasson U Cerebrospinal fluid matrix metalloproteinases and tissue inhibitor of metalloproteinases in combination with subcortical and cortical biomarkers in vascular dementia and Alzheimer’s disease J Alzheimers Dis 2011 5 665 676 21860087 10.3233/JAD-2011-110566

[B20] Sjogren M Blomberg M Jonsson M Wahlund LO Edman A Lind K Rosengren L Blennow K Wallin A Neurofilament protein in cerebrospinal fluid: a marker of white matter changes J Neurosci Res 2001 5 510 516 10.1002/jnr.1242 11746370

[B21] de Jong D Jansen RW Pijnenburg YA van Geel WJ Borm GF Kremer HP Verbeek MM CSF neurofilament proteins in the differential diagnosis of dementia J Neurol Neurosurg Psychiatry 2007 5 936 938 10.1136/jnnp.2006.107326 17314187 PMC2117885

[B22] Landqvist Waldo M Frizell Santillo A Passant U Zetterberg H Rosengren L Nilsson C Englund E Cerebrospinal fluid neurofilament light chain protein levels in subtypes of frontotemporal dementia BMC Neurol 2013 5 54 10.1186/1471-2377-13-54 23718879 PMC3671150

[B23] Madeddu R Farace C Tolu P Solinas G Asara Y Sotgiu MA Delogu LG Prados JC Sotgiu S Montella A Cytoskeletal proteins in the cerebrospinal fluid as biomarker of multiple sclerosis Neurol Sci 2013 5 181 186 10.1007/s10072-012-0974-4 22362332

[B24] Jeppsson A Zetterberg H Blennow K Wikkelso C Idiopathic normal-pressure hydrocephalus: pathophysiology and diagnosis by CSF biomarkers Neurology 2013 5 1385 1392 10.1212/WNL.0b013e31828c2fda 23486875

[B25] Tortelli R Ruggieri M Cortese R D’Errico E Capozzo R Leo A Mastrapasqua M Zoccolella S Leante R Livrea P Logroscino G Simone IL Elevated cerebrospinal fluid neurofilament light levels in patients with amyotrophic lateral sclerosis: a possible marker of disease severity and progression Eur J Neurol 2012 5 1561 1567 10.1111/j.1468-1331.2012.03777.x 22680408

[B26] Hagberg L Fuchs D Rosengren L Gisslen M Intrathecal immune activation is associated with cerebrospinal fluid markers of neuronal destruction in AIDS patients J Neuroimmunol 2000 5 51 55 10.1016/S0165-5728(99)00150-2 10626666

[B27] Mattsson N Bremell D Anckarsater R Blennow K Anckarsater H Zetterberg H Hagberg L Neuroinflammation in Lyme neuroborreliosis affects amyloid metabolism BMC Neurol 2010 5 51 10.1186/1471-2377-10-51 20569437 PMC2902447

[B28] Grahn A Hagberg L Nilsson S Blennow K Zetterberg H Studahl M Cerebrospinal fluid biomarkers in patients with varicella-zoster virus CNS infections J Neurol 2013 5 1813 1821 10.1007/s00415-013-6883-5 23471614

[B29] Jordanova A De Jonghe P Boerkoel CF Takashima H De Vriendt E Ceuterick C Martin JJ Butler IJ Mancias P Papasozomenos SCh Terespolsky D Potocki L Brown CW Shy M Rita DA Tournev I Kremensky I Lupski JR Timmerman V Mutations in the neurofilament light chain gene (NEFL) cause early onset severe Charcot-Marie-Tooth disease Brain 2003 5 590 597 10.1093/brain/awg059 12566280

[B30] Blennow K Wallin A Agren H Spenger C Siegfried J Vanmechelen E Tau protein in cerebrospinal fluid: a biochemical marker for axonal degeneration in Alzheimer disease? Mol Chem Neuropathol 1995 5 231 245 10.1007/BF02815140 8748926

[B31] Vanmechelen E Vanderstichele H Davidsson P Van Kerschaver E Van Der Perre B Sjogren M Andreasen N Blennow K Quantification of tau phosphorylated at threonine 181 in human cerebrospinal fluid: a sandwich ELISA with a synthetic phosphopeptide for standardization Neurosci Lett 2000 5 49 52 10.1016/S0304-3940(00)01036-3 10788705

[B32] Hansson O Zetterberg H Buchhave P Londos E Blennow K Minthon L Association between CSF biomarkers and incipient Alzheimer’s disease in patients with mild cognitive impairment: a follow-up study Lancet Neurol 2006 5 228 234 10.1016/S1474-4422(06)70355-6 16488378

[B33] Sarle WS SAS Institute Cubic Clustering Criterion 1983 Cary, NC: SAS Institute

[B34] Bjerke M Andreasson U Rolstad S Nordlund A Lind K Zetterberg H Edman A Blennow K Wallin A Subcortical vascular dementia biomarker pattern in mild cognitive impairment Dement Geriatr Cogn Disord 2009 5 348 356 10.1159/000252773 19864909

[B35] Gisslen M Krut J Andreasson U Blennow K Cinque P Brew BJ Spudich S Hagberg L Rosengren L Price RW Zetterberg H Amyloid and tau cerebrospinal fluid biomarkers in HIV infection BMC Neurol 2009 5 63 10.1186/1471-2377-9-63 20028512 PMC2807422

[B36] Krut JJ Zetterberg H Blennow K Cinque P Hagberg L Price RW Studahl M Gisslen M Cerebrospinal fluid Alzheimer’s biomarker profiles in CNS infections J Neurol 2013 5 620 626 10.1007/s00415-012-6688-y 23052602

[B37] Fagan AM Mintun MA Mach RH Lee SY Dence CS Shah AR LaRossa GN Spinner ML Klunk WE Mathis CA DeKosky ST Morris JC Holtzman DM Inverse relation between in vivo amyloid imaging load and cerebrospinal fluid Abeta42 in humans Ann Neurol 2006 5 512 519 10.1002/ana.20730 16372280

